# Aspirin in primary prevention and the risk of heart failure: a systematic review and meta‐analysis of controlled trials

**DOI:** 10.1002/ehf2.14269

**Published:** 2022-12-26

**Authors:** Ana Beatrice Magalhães de Oliveira, Beatriz Luchiari, Isabella Bonilha, Joaquim Barreto, Ana Claudia Cavalcante Nogueira, Guilherme Duprat Ceniccola, Carisi Anne Polanczyk, Andrei Carvalho Sposito, Luiz Sérgio Fernandes de Carvalho

**Affiliations:** ^1^ Laboratory of Data for Quality of Care and Outcomes Research (LaDa:QCOR) Universidade Católica de Brasília QS 07, Lote 01, Taguatinga Sul Brasília DF 71966‐700 Brazil; ^2^ Escola Superior de Ciências da Saúde (ESCS) Brasilia Brazil; ^3^ Laboratory of Atherosclerosis and Vascular Biology UNICAMP Campinas Brazil; ^4^ Aramari Apo Institute for Education and Clinical Research SCN Quadra 02 Bloco D, Entrada B Sala 1107 Brasília DF 70.712‐903 Brazil; ^5^ Nutrition Department, Hospital de Base do Distrito Federal Brasília Brazil; ^6^ Universidade Federal do Rio Grande do Sul Porto Alegre Brazil; ^7^ Clarity Healthcare Intelligence Marginal da Av Anhanguera, n 480 Jundiai SP 13087‐460 Brazil

The prevalence of heart failure (HF) worldwide nearly doubled from 33.5 million in 1990 to 64.3 million in 2017.^1^ Although the age‐standardized prevalence rate of HF has decreased by 20.3% in high‐income countries, it remained stable or increased in middle‐income countries (MIC) such as countries like India and China. Although the increased burden of obesity, diabetes and hypertension may justify this epidemiological scenario in MIC,^2^ a similar increase in these cardiometabolic factors does not seem sufficient to influence trends in high‐income countries. Exploring novel risk factors for HF in primary prevention setting may reveal new targets for policymakers. Mujaj et al.^3^ reported a cohort‐designed primary prevention (PP)‐based large‐scale study and showed a 26% increase in the risk of incident HF among aspirin users compared to non‐users. The authors did both multivariate and propensity score‐adjusted analyses to control for confounding factors, and pointed out for similar results.

These findings may suggest that aspirin use is a potential risk factor for incident HF. However, examining both the limitations of Mujaj's findings and the full body of evidence on aspirin in primary prevention is crucial before a definitive conclusion. At first, in this observational study it is not possible to avoid potential selection biases, that is, physicians tend to prescribe drugs believed to be life‐saving to more severely diseased individuals, and only randomized clinical trials (RCTs) can overcome this type of bias, producing balanced groups for comparison.^4^

Aiming to assess the incremental risk of HF among aspirin users versus non‐users, we searched for primary prevention‐based RCTs. We excluded literature reviews, editorials, conference records, congress abstracts or annals, in vitro studies, qualitative studies, cross‐sectional studies, case–control studies, systematic reviews and studies that used alternative treatments such as other types of anticoagulants. PubMed and Cochrane Library databases were used to search for original articles up to 8 December 2021, using MeSh terms and their synonyms. The search strategy is fully described in the supporting information. Sixty‐three citations were identified, all in PubMed. No duplicates were found. Three independent reviewers collected data from each article, firstly reading titles and secondly abstracts and keywords. The team used Rayyan QCRI application (Rayyan Systems Inc, MA, EUA) for managing duplicate files. Based on their abstracts, 1909 studies were excluded, and 401 with full text analysis were excluded because they did not mention primary prevention (232) or because they were not RCTs (169). Finally, four studies were included for meta‐analysis. The PRISMA flow chart that describes the process for study selection can be found in *Figure* S1. Regarding methodological quality and risk of bias, we applied the GRADE (Grading of Recommendations Assessment, Development and Evaluation) system to assess potential causes of bias (*Table* S1). To test for publication bias, we made funnel charts and performed the Egger test (*Table* S2 and *Figure* S2). We performed a meta‐analysis with random effects comparing aspirin users versus non‐users in selected RCTs. We assessed statistical heterogeneity between trials with *I* ^2^ statistic (with 95% CIs), which is derived from Cochran's Q [100 × (Q − df/Q)] and provides a measure of the proportion of overall variation that is attributable to between‐trial heterogeneity. In order to estimate the effect of the treatment, a two‐tailed *P*‐value <0.05 was considered statistically significant. The extracted data were analysed using R Studio v.1.1.463 with R language v.4.0.1 and *metaviz*/*metafor* packages.

In total, four RCTs were included with 40 418 patients. Of these trials, two reported incident HF as an outcome and two reported hospitalizations due to HF. To extend statistical power, we pooled both outcomes to estimate the risk of HF in individuals using aspirin. As shown in *Figure* *1*, homogeneously across trials, we found no association between aspirin use and a risk of HF (odds ratio 0.93; confidence interval of 0.77 to 1.13, *P* for difference of 0.502, *I* ^2^ = 5.57%, *P* for heterogeneity of 0.494). These findings suggest to be less than 1% the probability of aspirin use to increase the risk of HF by 15% or more. This analysis reached a statistical power of 80.78%.^4^ However, a couple of limitations must be acknowledged in our study: (i) information regarding types of HF (with reduced or preserved ejection fraction) was not clear in the selected studies; (ii) it is also essential to mention that due to a certain degree of heterogeneity in our sample, there may be some subgroups of interest to be analysed in future studies, such as diabetic individuals or the elderly.

**Figure 1:**
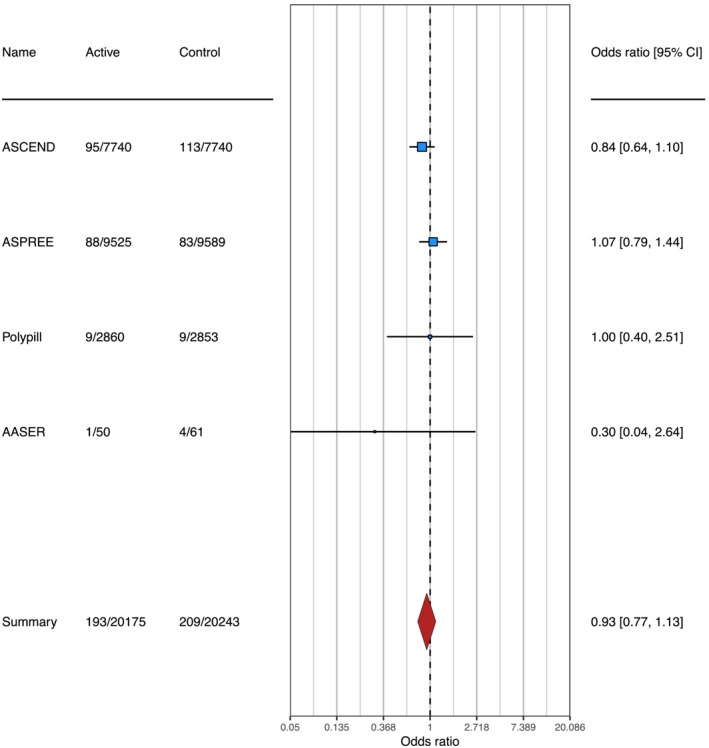
Forrest plot for incident heart failure among four trials included after systematic review. I^2^ = 2% (confidence interval 95%: 0 to 15%, *P* = 0.782).

Although the hypothesis of an association between aspirin therapy and HF has been recently raised,^3^ most of the mechanisms supporting this relationship are based on unestablished grounds. Aspirin use, for example, was associated with reduced renal function,^5^, ^6^ but this effect was mild and reported in short‐term follow‐ups (<2 weeks). The reduction of platelet adhesion with aspirin could potentially contribute to the leakage of neovascular vessels.^7^ Nevertheless, the demonstration of this effect is pending. Chronic use of aspirin may be associated with iron deficiency^8^ and, by this way, may deteriorate the clinical course of individuals with established HF.^9^ The triggering of HF, however, is unlikely to result exclusively due to anaemia or iron deficiency except in cases of severe and prolonged anaemia.^10^

The results of this systematic review and meta‐analysis indicates that even if the aforementioned mechanisms are confirmed, their clinical relevance of the association between aspirin use and HF is limited. It is important to stress that our results do not favour or could be used to recommend aspirin use in primary prevention. The key message is that a relationship between chronic aspirin use and the risk of HF in primary prevention is unlikely to be of any clinical significance or be responsible for HF epidemiologic trends. Also, in terms of primary prevention of cardiovascular events, aspirin is not recommended in the 2021 ESC Prevention Guidelines^11^ due to its lack of efficacy in face of the increased risk of bleeding. According to recent data, there could be however subgroups where aspirin in primary prevention of cardiovascular events could be beneficial and the risk of HF and bleeding has to be put into perspective.^12^

## Funding

This work was supported by grant 2019/09068‐3 from São Paulo Research Foundation (FAPESP), grant 371/2021 from Federal District Research Foundation (FAPDF) and grants 437413/2018‐7 and 301465/2017‐7 from the Brazilian National Research Council (CNPq). The funder had no role in the design and conduct of the study; collection, management, analysis, and interpretation of the data; preparation, review, or approval of the manuscript; and decision to submit the manuscript for publication.

## Supporting information


**Data S1.** Supporting Information.

**Table S1.** Risk of bias assessment of included randomized controlled trials.
**Table S2.** Regression tests for funnel plot asymmetry.
**Table S3.** Baseline characteristics.
**Figure S1.** PRISMA Flow diagram detailing the selection process applied.
**Figure S2.** Funnel plot.
