# Supplementary material for: Aspirin in primary prevention and the risk of heart failure: a systematic review and meta‐analysis of controlled trials
Source: ESC Heart Fail. 2022 Dec 26;10(2):1488–91. doi: 10.1002/ehf2.14269 (PMC10053165; doi:10.1002/ehf2.14269)
Supplement: Supplementary file 1 — Data S1. Supporting Information. [file EHF2-10-1488-s002.doc]

**Systematic Review Protocol**

Title:

| **Aspirin in Primary Prevention and the Risk of Heart Failure: A Systematic Review and Meta-Analysis of Randomized Controlled Trials** |
| --- |

Keywords:

| Aspirin, Primary Prevention, Heart Failure, Systematic Review. |
| --- |

Actual start date/ Anticipated completion date:

| **From: December 2021** | **To: January 2022** |
| --- | --- |
| **Search: 08/12/2021** | |

Funding:

| This work was supported by grant 2019/09068-3 from São Paulo Research Foundation (FAPESP), grants 437413/2018-7 and 301465/2017-7 from the Brazilian National Research Council (CNPq). The funder had no role in the design and conduct of the study; collection, management, analysis, and interpretation of the data; preparation, review, or approval of the manuscript; and decision to submit the manuscript for publication. |
| --- |

Conflict of interest:

| No conflict of interest |
| --- |

**Research Team members and contact information**

|  | **Name** | **Affiliation** | **Email** | **Contributions** |
| --- | --- | --- | --- | --- |
| **First author** | Ana Beatrice Magalhães de Oliveira | Foundation of Education and Research in Health Sciences – FEPECS, Brasilia Brazil | beatricemoliveira@gmail.com | 1R |
| **Second reviewer** | Beatriz Luchiari | Laboratory of Atherosclerosis and Vascular Biology UNICAMP, Campinas, SP, Brazil | bialuchiari@hotmail.com | 2R |
| **Third reviewer** | Isabella Bonilha | Laboratory of Atherosclerosis and Vascular Biology UNICAMP, Campinas, SP, Brazil | isaoliveira.ib@gmail.com | 3R |
| **Expert (field)** | Ana Claudia Cavalcante Nogueira; Andrei Carvalho Sposito; Carisi Anne Polanczyk; Joaquim Barreto | Laboratory of Atherosclerosis and Vascular Biology UNICAMP, Campinas, SP, Brazil | nogueira.anaclaudia@gmail.com, andreisposito@gmail.com | E |
| **Sub-coordinator (expert in SR methodology)** | Guilherme Duprat Ceniccola | SES-DF | guiduprat@gmail.com | C |
| **Coordinator**  **(expert in SR methodology)** | Luiz Sergio F de Carvalho | Foundation of Education and Research in Health Sciences – FEPECS, Brasilia Brazil | luizsergiofc@gmail.com | C |

1R=First reviewer (Study conceptualization and design/ Search and selection/ Data collection/ Data analysis/ Manuscript preparation). 2R=Second Reviewer (Search and selection/ Data collection/ Data analysis/ Manuscript preparation). 3R=Third Reviewer (Data analysis). E=Expert (Study conceptualization and design/ Data analysis). C=Coordinator (Study conceptualization and design/ Data analysis).

All authors: Review of the manuscript.

**Methods**

Question:

| **Is the use of aspirin in primary prevention associated with the incidence of heart failure in randomized controlled trials?** |
| --- |

**Inclusion criteria**

We will include clinical studies (RCTs) showing patients in primary prevention for cardiovascular events who were treated with aspirin compared with those not treated (control group), evaluating the incidence of heart failure in this population.

| **PICOS** |  | **Key Words** |
| --- | --- | --- |
| **Types of participants (P)** | Patients in primary prevention | "Primary Prevention"[Mesh] OR (Disease Prevention, Primary) OR (Disease Preventions, Primary) OR (Primary Disease Prevention) OR (Primary Disease Preventions) OR (Prevention, Primary) OR (Primordial Prevention) OR (Preventions, Primordial) OR (Primordial Preventions) OR (Prevention, Primordial) |
| **Types of interventions (I)** | Aspirin | "Aspirin"[Mesh] OR (Acetylsalicylic Acid) OR (Acid, Acetylsalicylic) OR (2-(Acetyloxy)benzoic Acid) OR (Acylpyrin) OR (Aloxiprimum) OR (Colfarit) OR (Dispril) OR(Easprin) OR (Ecotrin) OR (Endosprin) OR (Magnecyl) OR (Micristin) OR (Polopirin) OR (Polopiryna) OR (Solprin) OR (Solupsan) OR (Zorprin) OR (Acetysal) |
| **Comparison (C)** |  |  |
| **Types of outcome measures (O)** | Heart failure | "Heart Failure"[Mesh] OR (Cardiac Failure) OR (Heart Decompensation) OR (Decompensation, Heart) OR (Heart Failure, Right-Sided) OR (Heart Failure, Right Sided) OR (Right-Sided Heart Failure) OR (Right Sided Heart Failure) OR (Myocardial Failure) OR (Congestive Heart Failure) OR (Heart Failure, Congestive) OR (Heart Failure, Left-Sided) OR (Heart Failure, Left Sided) OR (Left-Sided Heart Failure) OR (Left Sided Heart Failure) |
| **Types of studies (S)** | RCT’s | "Randomized Controlled Trial" OR "Clinical Trials, Randomized" OR "Trials, Randomized Clinical" OR "Controlled Clinical Trials, Randomized" |

**Exclusion criteria**

Phase 1 Abstract and title

1. Literature review;
2. Editorials∕Letters;
3. Conferences, Summaries and Annals;
4. In vitro studies;
5. Studies of animal models;
6. Qualitative studies;
7. Cross-sectional studies;
8. Case-control studies;
9. Systematic reviews, overview and scoop reviews;
10. Alternative studies using other types of anticoagulants.

**Phase 2 Full paper**

1. Studies not including Aspirin

**Search strategy (PubMed): Tested on 2021-12-12**


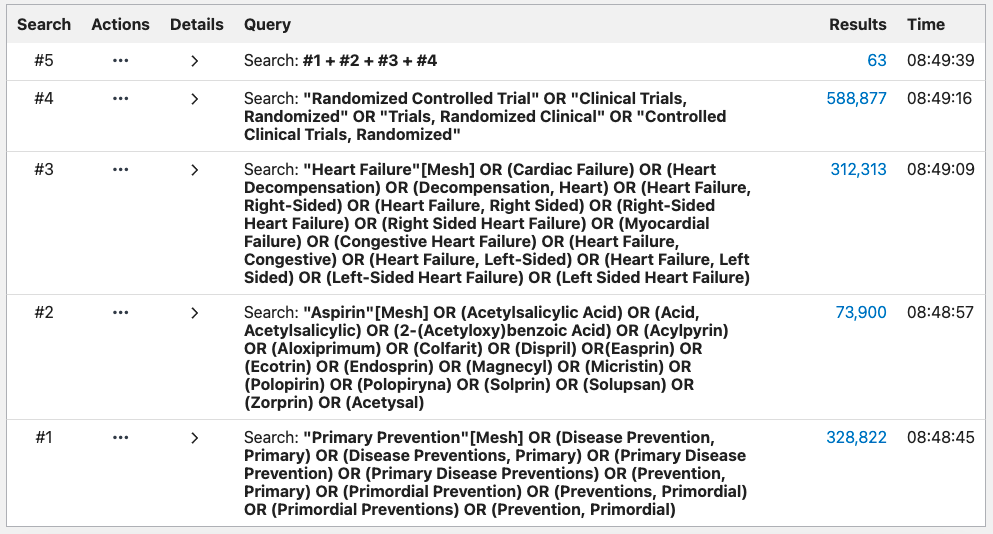


**Search methods for identification of studies**

**Databases:**

1. PubMed ( X )

2. Cochrane ( X )

3. LILACS ( )

4. Web of Science ( )

5. Scopus ( )

6. EMBASE ( )

7. Other ____________

**Additional Literature**

1. Google Scholar web search( )

2. Hand searches of bibliographies from included studies( )

3. Experts ( )

4. JSTOR ( )

5. Open Grey ( )

Risk of bias assessment tool

| The Cochrane Collaboration’s tool for assessing risk of bias in randomized trials - BMJ 2011;343:d5928doi: 10.1136/bmj.d5928 |
| --- |

Analysis of subgroups or subsets (Give any planned exploration of subgroups or subsets within the review. ‘None planned’ is a valid response if no subgroup analyses are planned)

| None planned |
| --- |

Type of review

( )Epidemiologic

( ) Prevention

(x) Intervention

( ) Diagnostic

( ) Prognostic

Previous systematic reviews about the same subject: ( ) yes (x) no
