# Supplementary material for: Aspirin in primary prevention and the risk of heart failure: a systematic review and meta‐analysis of controlled trials
Source: ESC Heart Fail. 2022 Dec 26;10(2):1488–91. doi: 10.1002/ehf2.14269 (PMC10053165; doi:10.1002/ehf2.14269)
Supplement: Supplementary file 2 — Table S1. Risk of bias assessment of included randomized controlled trials. Table S2. Regression tests for funnel plot asymmetry. Table S3. Baseline characteristics. Figure S1. PRISMA Flow diagram detailing the selection process applied. Figure S2. Funnel plot. [file EHF2-10-1488-s001.docx]

**Supplemental Table S1.** Risk of bias assessment of included randomized controlled trials.

|  | Risk of Bias | | | | | | |
| --- | --- | --- | --- | --- | --- | --- | --- |
| *First author* | *Random sequence generation* | *Alocation Concealment* | *Blinding of patients, personnel* | *Blinding of outcome assessors* | *Incomplete outcome data* | *Selective outcome reporting* | *Others* |
| 1. AASER | Low | Unclear | Unclear | Low | Unclear | Low | Low |
| 2. ASPREE | Low | Low | Low | Low | Unclear | Low | Low |
| 3. ASCEND | Low | Low | Unclear | Unclear | Low | Low | Low |
| 4. Polypill | Low | Low | Unclear | Low | Low | Low | Low |

**Supplemental Table S2**. Regression tests for funnel plot asymmetry

|  | | |
| --- | --- | --- |
|  | weighted regression with multiplicative dispersion | mixed-effects meta-regression model |
| **Hospitalizations due to heart failure** | t = -0.64, p = 0.58 | z = -0.67, p = 0.50 |

**Supplemental Table S3.** Baseline characteristics

| **Study** | **Active**  **arm** | **Control**  **arm** | **Sample**  **size** | **Follow-up time**  **(years)** | | **Women**  **(%)** | **age, years**  **(mean±SD)** | **Prior**  **diabetes**  **(%)** | **Prior Hypertension (%)** | **Prior**  **Heart Failure**  **(%)** |
| --- | --- | --- | --- | --- | --- | --- | --- | --- | --- | --- |
| AASER | Aspirin | Standard  care | 111 | 5.4 | 32.43 | | 68±8.3 | 39 | 90 | 10 |
| ASPREE | Aspirin | Placebo | 19114 | 4.7 | 56.41 | | 64±8.1 | 19.4 | 13.3 | - |
| ASCEND | Aspirin | Placebo | 15480 | 7.4 | 37.44 | | 63.2±9.2 | 94.1 | 61.6 | - |
| Polypill | Polypill (simvastatin, ramipril, antenolol, hydrochlorothiazide) | Placebo | 5713 | 4.6 | 52.95 | | 63.9±7.8 | 36.7 | 83.8 | 0.4 |

**Supplemental Figure S1.** PRISMA Flow diagram detailing the selection process applied.


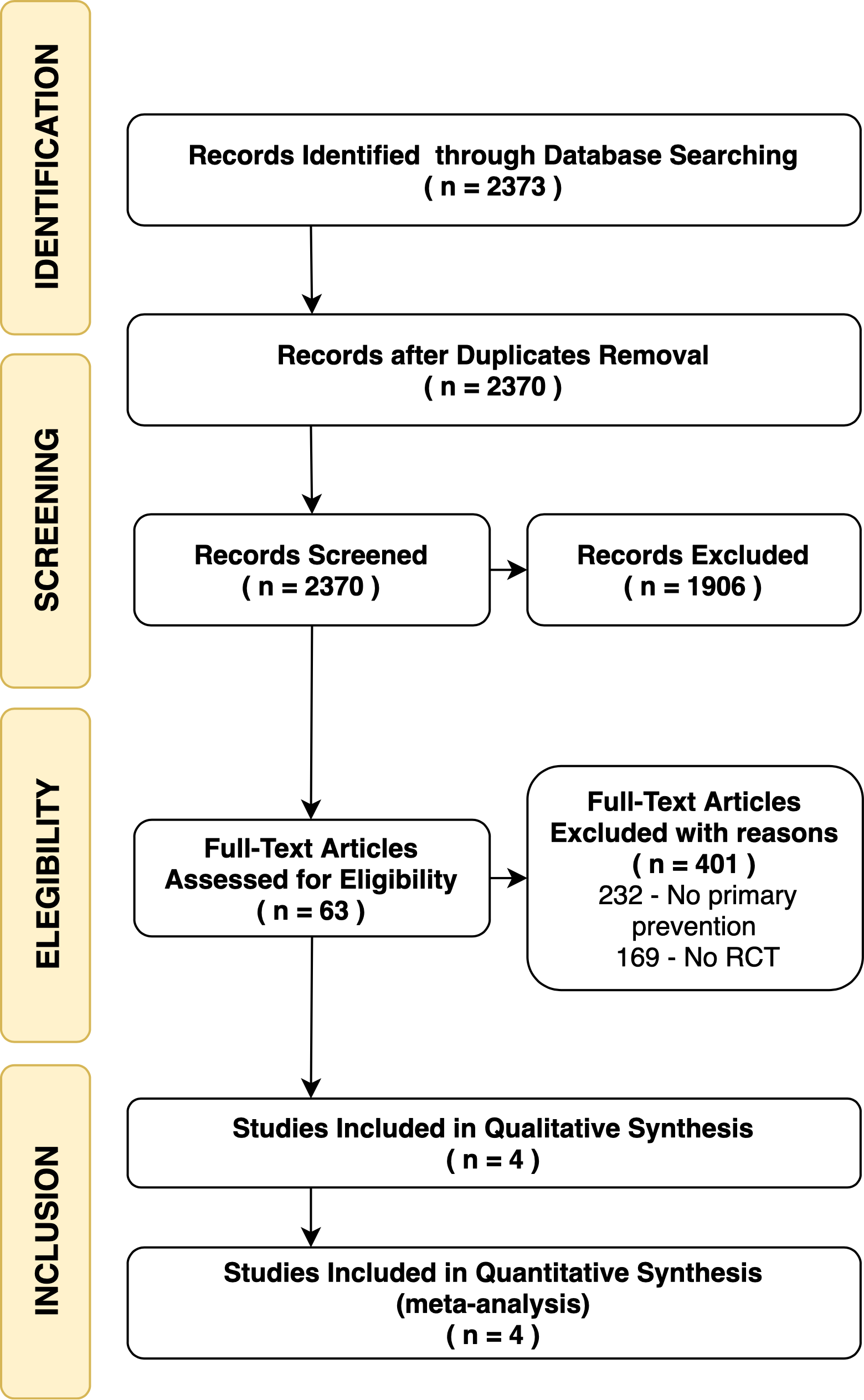


**Supplemental Figure S2.** Funnel plot
